# Development of intelligent robots in the wave of embodied intelligence

**DOI:** 10.1093/nsr/nwaf159

**Published:** 2025-04-25

**Authors:** Weijie Zhao, Ye Yuan

**Affiliations:** NSR, Beijing; School of Mechanical Science and Engineering, Huazhong University of Science and Technology, China

## Abstract

On January 28, 2025, 24 humanoid robots from Unitree Ltd. performed alongside human dancers on the Spring Festival Gala of China Central Television. This performance captivated tens of millions of viewers and sparked widespread discussion across China. As Elon Musk ambitiously unveiled Tesla's plans for intelligent robots and various humanoid robot prototypes began appearing on the streets of major cities, embodied intelligence has emerged as the new frontier in science and technology. But what do terms like ‘end-to-end embodied large model’ and ‘general intelligent robot’ truly mean? Is the scientific foundation of embodied intelligence robust? How far can the embodied intelligence industry, particularly the intelligent robot sector, progress? Will ‘one robot per household’ become a reality within a few years, fundamentally transforming human production and lifestyle? In a timely panel discussion chaired by Professor Han Ding of Huazhong University of Science and Technology, seven leading researchers in embodied intelligence and robotics gathered to explore these questions. They delved into the scientific basis, technical roadmap, current developments, and major challenges in the field.

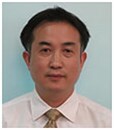

Qijun Chen

Professor, School of Electronic and Information Engineering, Tongji University, China

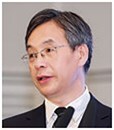

Yongchun Fang

Vice President, Nankai University, China; Professor, School of Artificial Intelligence, Nankai University, China

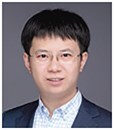

He Wang

Assistant Professor, School of Computer Science, Peking University, China; Founder and CTO, Beijing Galbot Co., Ltd., China

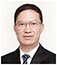

Yaonan Wang

Professor, School of Electrical and Information Engineering, Hunan University, China

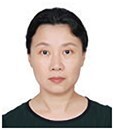

Rong Xiong

Professor, School of Control Science and Engineering, Zhejiang University, China

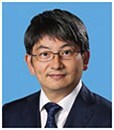

Jing Xu

Associate Professor, Department of Mechanical Engineering, Tsinghua University, China

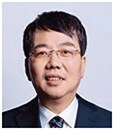

Han Ding (Chair)

Professor, School of Mechanical Science and Engineering, Huazhong University of Science and Technology, China

## Teaser

Seven leading researchers in embodied intelligence and robotics gathered to talk about the scientific basis, technical roadmap, current developments, and major challenges in the field.

## AI IS MAKING ROBOTS INTELLIGENT AND GENERAL

**Ding:** Embodied intelligence is a broad concept. Any intelligent system with a physical body and capable of perceiving and interacting with the external environment falls under this category, including but not limited to intelligent cars, intelligent robots, and smart home devices.

Today's discussion will focus on intelligent robots. The robot is the flagship carrier of embodied intelligence, and intelligent robots have reached the brink of technological breakthroughs and real-world applications.

Let's begin by discussing how artificial intelligence (AI) is enabling and transforming robotics.

**Xu:** Traditional robots rely on pre-programmed models and fixed routines to perform specific tasks. For instance, industrial robots are programmed for repetitive actions and can process only specific parts. They lack flexibility and cannot adapt to complex, dynamic environments.

In contrast, one of the key characteristics of AI is its strong generalization ability, which can enable robots to handle new scenarios and tasks intelligently. With AI methods introduced, robots can adjust their behaviors autonomously in changing scenarios, exhibiting greater adaptability and flexibility. In essence, AI is making robots intelligent and general.

**H Wang:** The embodied intelligence community is currently focused on the development of two types of models. The first is ‘vision-driven embodied small models’, which are each designed for a specific kind of task. For example, we can train a model to control a dexterous hand to grasp various objects, regardless of their shape, position, or orientation.

The second is the ‘end-to-end embodied large model’, which aims to handle general tasks with a single large model. ‘End-to-end’ refers that the model will bypass the traditional robot workflow of perception-decision-planning-execution, and directly links sensor inputs (the input end) to robot actions (the output end).

The end-to-end approach gained popularity mainly due to its success in autonomous driving. Tesla's end-to-end autonomous driving system can directly output the instantaneous rotation angle of the steering wheel and the working degree of the accelerator or the brake, from the sensors’ inputs. Several Chinese smart car companies have also developed end-to-end models with impressive results.

It is now widely accepted that end-to-end is the ultimate solution for autonomous driving, so people are beginning to think that the end-to-end embodied model, coupled with a humanoid robot as the body, may be a competitive candidate of the ultimate solution for a general intelligent robot.

The end-to-end embodied large model is often envisioned as a Vision-Language-Action (VLA) model, which would enable robots to understand human language commands and relying upon visual signal input to perform corresponding tasks, which can be grasping objects, twisting bottle caps, cooking and so on.

The VLA concept was first proposed by Google when it released the RT-2 (Robotics Transformer 2) model in 2023. Many groups are now developing VLA models. Google has proposed a large-scale VLA with 55B parameters, and our company Galbot has developed an end-to-end embodied model with 2.7B parameters.

Despite the current data limitations, I believe that the emergence point of the embodied large model will soon be reached, and the large model will unify the small models, bringing general intelligent humanoid robots to life. These robots will understand natural language commands, learn new tasks from minimal demonstrations, and operate seamlessly in open environments.

People are beginning to think that the end-to-end embodied model, coupled with a humanoid robot as the body, may be a competitive candidate of the ultimate solution for a general intelligent robot.

—He Wang

**Chen:** After the introduction of intelligent technologies, one critical question to consider is: What should the future architecture of robotic systems look like? I believe that the rapid advancement of intelligent technologies is reshaping the theoretical foundations and architectural paradigms of robotic systems. As Dr. Wang pointed out, from the perspective of control architecture evolution, the traditional cascaded closed-loop framework of perception-decision-planning-execution is being redefined by end-to-end learning models, forming a new ‘outer loop’ structure that directly links perception to action. This new ‘perception-action’ outer loop fundamentally establishes a high-dimensional nonlinear mapping from multimodal inputs to motor torque outputs, enabling robot motion patterns that are jointly driven by tasks, environments, and physical embodiments. This necessitates moving beyond classical control theory based on differential equations and developing new system methodologies that can accommodate data-driven paradigms.

I foresee a dual transformation in future system architectures.

First, at the control theory level, a dual-track framework integrating physics-based and data-driven approaches will emerge. Classical control methods will be retained at the low-level execution layer to ensure fundamental stability, while the high-level decision-making layer will leverage probabilistic learning strategies for robust control in complex scenarios. We need to explore new mathematical tools that organically integrate the generalization capability of neural networks with Lyapunov stability analysis—for instance, by constructing energy functions to constrain the weight update directions of neural networks.

Second, at the system topology level, the trend of brain-body separation is driving the dynamic reconfiguration of computational resources. Existing studies indicate that adopting a federated reinforcement learning framework and offloading computational loads to the cloud can significantly enhance the efficiency of updating parameters in on-board controllers. However, it may be necessary to establish new evaluation metrics to quantify the impact of network latency on system stability, thereby optimizing network communication demands, dynamically allocating and scheduling cloud and local computing resources, and ultimately achieving optimal system performance.

The rapid advancement of intelligent technologies is reshaping the theoretical foundations and architectural paradigms of robotic systems.

—Qijun Chen

## SPECIALIZED INTELLIGENT ROBOTS: RAPID DEVELOPMENT

**Ding:** Small embodied intelligence models are already empowering specialized intelligent robots. Could you share recent progress from your teams?

**Chen:** My laboratory, the Robotics and Artificial Intelligence Laboratory (RAIL), has built a stable research team over more than 30 years of development. We are equipped with industrial robots, service robots, humanoid robots, autonomous vehicles, high-performance servers, and various testing and validation instruments, equipment, and software tools. From early research on traditional robotic control to end-to-end perception and decision-making, and now to large-model-driven paradigms, we have consistently focused on using AI algorithms to solve real-world robotic challenges.

Currently, our team is developing intelligent robots for disaster responses. While robots have been used in disaster responses for some time, they still lack sufficient flexibility and autonomy. We are exploring ways to integrate human-like abilities—such as processing visual and language cues—to handle unexpected and extreme situations. By enabling disaster response robots to think and adapt like humans, we aim to enhance their ability to make rapid autonomous decisions based on real-time conditions at disaster sites, ultimately improving their effectiveness in real-world rescue operations.

**Fang:** We have developed snake-like robots for underground exploration and other tasks. Empowered by AI, these robots can autonomously perform multi-modal perception, route planning, and movement control. They have been field-tested in Xuzhou and other locations.

We are also collaborating with Xuzhou Heavy Machinery to integrate large language models (LLMs) and visual models into cranes and other construction machinery, enabling them to understand human language commands and perform complex tasks.

**Xiong:** Visual servo control, a fundamental capability of robots to carry out a certain operation after recognizing a certain visual signal, has been optimized using machine learning. Our system can effectively recognize visual targets with different characteristics, and can be adapted to different scenarios. Specifically, the camera can be put anywhere, even not on the robot, and we do not need to precisely calibrate the camera before the system can start to work.

For mechanical servo control, we have advanced the classic Peg-in-Hole task, which means to insert one object (peg) into another object (hole). Through imitation and reinforcement learning, our system can achieve a 99.99% success rate and <0.1 mm tolerance for new target shapes, with one-hour's training on the real machine. These parameters are world-leading and are superior to DeepMind's similar systems. This system has been applied to Huawei's production lines since May 2024.

**H Wang:** During my doctoral studies, I focused on 3D vision for robotics, including 6D pose estimation of an object that is known to belong to a certain category, such as a cup, but does not have a CAD model. Back at Peking University since 2021, we extended this research to downstream motion control. In 2022, we won the global championship of the unmarked track of the ICLR Robot ManiSkill Challenge. The challenge set up four tasks, including opening drawers, opening cabinet doors, pushing chairs and moving buckets, and each task included up to dozens of different manipulation targets.

We also developed a generalized reinforcement learning strategy for dexterous hand grasping. This work was elected a best paper candidate of ICCV.

**Ding:** Recent years have seen rapid advancements in dexterous hands, with significant improvements in performance and rapid reduction of cost. The human hand is a very delicate machine. If we can revolute both the hardware and software of the dexterous hands, and make them able to accomplish various delicate operations that can only be done by human hands now, that would mark a major milestone of embodied intelligence.

## GENERAL INTELLIGENT ROBOTS: IS HUMANOID THE ANSWER?

**Ding:** Dr. Wang just mentioned that the carrier of the upcoming end-to-end general embodied intelligence large model is likely to be humanoid robots. In recent years, humanoid robots have garnered great attention, with their motion performance videos amazing everyone.

Professor Xiong, your team is one of the earliest and most accomplished in China working on humanoid robots. Could you share your insights on their development?

**Xiong:** Humanoid robots are indeed a hot topic right now. Many research groups, including Professor Ding's, have developed humanoid robots with diverse characteristics.

It can be said that humanoid robots are the best carriers of embodied intelligence, but are also their greatest challenge. They are the best carriers because their generalized structure enables them to perform a wide range of tasks in various scenarios.

**Figure 1:**
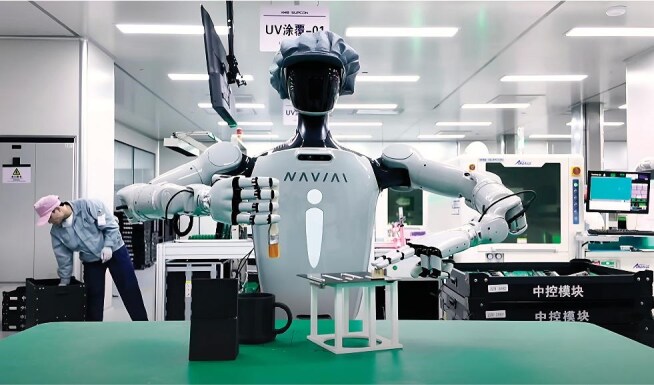
Humanoid robot NAVIAI is performing a printed circuit board (PCB) coating task via collaboration of dual-arms and hands. *(Photo provided by Prof. Rong Xiong)*

However, their extreme complexity—with numerous degrees of freedom and a vast array of sensors—makes it a monumental challenge to effectively control such a body to accomplish complex and delicate practical tasks.

The introduction of AI technologies has opened up tremendous opportunities for humanoid robots. Unlike non-embodied LLMs, which learn from text and other virtual data to develop reasoning abilities, embodied intelligence models learn through interactions between the robot and its environment to form capabilities of perception, planning, and decision-making.

A prime example is how humanoid robots learn to walk. By leveraging simulated and real-world data of robot motion and environmental interactions, techniques like imitation learning and reinforcement learning enabled the robots to learn how to walk like a human. The robustness and other parameters achieved through these methods have far surpassed traditional control approaches.

With the basic ability of walking now mastered, the next step for humanoid robots is to acquire more practical skills. One of our team's current projects focuses on coordinating the movements of the upper and lower limbs to maintain balance while the upper limbs are performing tasks.

Humanoid robots are the best carriers of embodied intelligence, but are also their greatest challenge.

—Rong Xiong

**Y Wang:** Humanoid robots are advancing rapidly and have reached a critical stage in transitioning from laboratory research to practical applications. However, despite the impressive videos showcasing their movement skills, experts have recognized that there is still a significant gap between current technology and our expectations for these robots to perform practical tasks.

**Ding:** Exactly. The primary purpose of creating robots is to replace human labor, thereby increasing production efficiency and making our lives more convenient. Over the past few decades, industrial robots have replaced a large number of welders and spray-painting workers on automobile production lines, significantly reducing labor costs and making cars more affordable for general consumers. Without these industrial robots, most people would not be able to afford cars for their daily use.

Looking to the future, we expect general intelligent humanoid robots to replace skilled human workers across various production lines, performing tasks that are far more delicate than welding or spray-painting. While this transition will not happen overnight, the current progress in embodied intelligence gives us hope that it will be achieved through a gradual evolution of technology.

We expect general intelligent humanoid robots to replace skilled human workers across various production lines, performing tasks that are far more delicate than welding or spray-painting.

—Han Ding

## THE CHALLENGES

### The gaps between AI and robotics

**Fang:** The powerful reasoning and interactive capabilities of AI have the potential to bring significant advancements to robotics. However, it is important to recognize that AI and robotics are distinct fields with different characteristics. Current LLMs, while impressive, fall short in meeting certain critical requirements for robots, such as real-time performance, accuracy, and reliability.

Unlike LLMs, which typically do not demand high real-time performance, robots operate in dynamic environments where they cannot pause to deliberate on their next move. To enhance the real-time capabilities of embodied intelligence, it may be necessary to miniaturize the large models when deploying them locally. Techniques such as knowledge distillation can be employed to improve response speed and ensure timely decision-making.

One notable characteristic of LLMs is their ability to tolerate a degree of uncertainty. When prompted with a question, these models can generate a range of similar yet varied responses. However, robotic operations demand precision. Embodied intelligence must provide an ‘optimal solution’ to ensure effective and accurate task execution.

Security and reliability are also paramount for robots. Current LLMs cannot guarantee that their outputs are always correct and will not cause problems in the real world. To address this, researchers have developed methods to enhance the reliability of embodied intelligence. For instance, the introduction of space-time constraints and temporal logic can help ensure that robotic operations adhere to physical laws. By forcing the robots’ operation to meet these physical constraints—including constraints of different levels, from very tight ones to relatively loose ones—the safety and reliability of the system can be improved.

Current LLMs, while impressive, fall short in meeting certain critical requirements for robots, such as real-time performance, accuracy, and reliability.

—Yongchun Fang

### Data shortage and the potential solutions

**H Wang:** For model development, the primary bottleneck at present is the lack of training data. There are two main approaches to address this issue: to collect data through human teleoperation, or to generate a large volume of virtual data using synthetic data generation techniques.

Tesla has opted for the first approach, employing thousands of people to conduct large-scale teleoperation. Some companies in China are also following this path. However, I personally believe that it's difficult to collect sufficient data for training a large embodied model relying solely on teleoperation. While human teleoperation may be able to generate millions of pieces of data, training a robust general large embodied model may require billions or even tens of billions of pieces of data.

In the field of autonomous driving, real-world driving data from users can effectively support the continuous evolution of models. However, for intelligent robots, we cannot release a semi-finished product to users and expect them to generate data back for the robot companies.

At Galbot, we believe that simulation and synthetic data techniques are keys to solving this problem. We are confident that these technologies can generate enough data to accomplish the pre-training of a general embodied large model. Galbot has released the world's first large-scale synthetic dataset for dexterous hand grasping, along with an end-to-end VLA model trained on this dataset. This system can accept human language instructions to grasp a wide variety of objects, whether it's a stapler, an elephant, or even an uncommon item like a hinge line.

**Figure 2:**
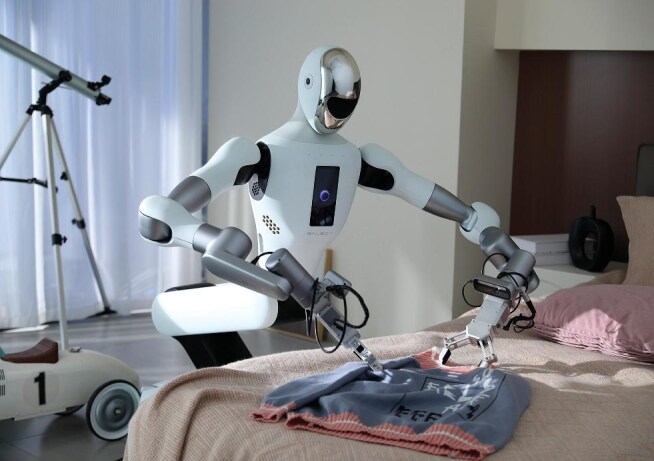
Galbot robot uses coordinated dual arms to hang clothes on a hanger. *(Photo provided by Galbot)*

Of course, synthetic data methods are not without limitations. While our ability to generate visual data is relatively mature, producing data for other modalities—such as haptic, temperature, and sound data—remains a significant challenge.

**Xu:** In the training process of an embodied intelligence model, a critical step is Sim2Real transfer—that is, transferring the skills learned from simulation data into real-world applications. To enhance Sim2Real capabilities, we need to minimize the gap between simulated and physical environments and ensure that the generated data align with physical laws. Currently, AI cannot fully comprehend physical laws, so it is essential to introduce relevant constraints.

My research group has made progress in this area by developing Sim2Real platforms for visual and tactile sensors. These platforms have significantly improved the efficiency of transfer from simulation to real-world scenarios.

### Tactile perception is a difficulty

**Ding:** Robots require multimodal perception and interaction with their environment. Vision is the most critical modality, while other senses such as touch and sound are also essential. For instance, the current Da Vinci surgical robot primarily relies on visual signals, whereas human surgeons depend heavily on mechanical tactile feedback through their hands to perform precise surgeries. If tactile signals can be effectively integrated in the future, I believe the performance of surgical robots will reach a new level of excellence.

**Xiong:** To execute fine manipulations, robots must inevitably make direct contact with their targets and adjust their operations based on tactile feedback. However, the simulation authenticity of current tactile simulation platforms, including NVIDIA's Isaac and the Genesis platform developed by Carnegie Mellon University in collaboration with over 20 institutions, remains limited. This poses a significant constraint on robots’ ability to learn to perform delicate tasks effectively.

**Xu:** Enhancing the tactile perception capabilities of embodied intelligence requires not only improvements in modeling but also advancements in hardware. My group is currently developing tactile sensors and working to build a tactile perception system with high resolution, rapid frequency response, and good real-time processing capabilities.

### Improve the body

**Ding:** As Professor Chen mentioned, the final operation of robots still relies heavily on electric motors. To date, electric motors remain the most efficient and reliable actuators for robots, and we have yet to find a viable alternative. While new actuation technologies, such as pneumatic muscles, are frequently featured in top-tier journals, translating these innovations from research papers to practical applications remains a significant challenge. Even small advancements in the fundamental technologies of the robot bodies are exceptionally difficult to achieve.

**Xu:** Most robots are currently powered by electric motors. However, in nature, the movement systems of animals are remarkably diverse and fascinating. For example, an octopus uses the soft, flexible properties of its tentacles to grasp objects. These tentacles can adapt to the shape of an object without the need of complex calculations or precise control of limb joints. Similarly, the human hand has a soft surface that allows it to conform to the shape and texture of objects being grasped.

I believe we should draw inspiration from nature's soft designs when developing robots, making them more flexible and versatile. However, such soft designs often demand advanced tactile perception and control capabilities, which brings us back to the tactile challenges we discussed earlier.

**Y Wang:** Without a well-designed body, embodied intelligence cannot fully realize its potential. Therefore, research and innovation in robotic physical systems must remain a top priority and should never be neglected.

Most robots are currently powered by electric motors. However, in nature, the movement systems of animals are remarkably diverse and fascinating.

—Jing Xu

## PERSPECTIVES TO THE FUTURE

### Interdisciplinary collaboration is essential

**Y Wang:** In the field of embodied intelligence, interdisciplinary collaboration is essential. New discoveries and technologies from brain and cognitive science, materials science, information science, and other fields must be rapidly integrated into this domain to drive progress.

**Xu:** I completely agree with the importance of interdisciplinary research. For instance, the way animals generate intelligence and make decisions is fundamentally different from the current AI systems. Nematodes, for example, can perform highly complex intelligent behaviors with as few as ∼300 neurons. In contrast, AI systems require vast numbers of artificial neurons and immense GPU computing power to make decisions, making them far less efficient than biological intelligence in terms of energy consumption and responsiveness.

I have long been fascinated by these phenomena and am currently collaborating with neuroscientists at Tsinghua University to study how animals make decisions and learn new skills. I believe that a deeper understanding of biological intelligence and other natural mechanisms will significantly advance the development of embodied intelligence.

**Ding:** The mechanisms underlying biological intelligence are incredibly complex. It may take scientists considerable time to fully understand and imitate these mechanisms. However, even if we focus on embodied intelligence technologies with near-term application potentials, the integration of expertise from diverse fields is also crucial. Breakthroughs will only be achieved when talents from computer science, control engineering, mechanics, materials science, brain science, and other disciplines come together to work toward a shared application goal.

### China's chance in the field

**Y Wang:** I sincerely hope to see more groundbreaking achievements from China in the field of embodied intelligence. China has already established itself as one of the leaders in the emerging fields such as digital transformation, new energy vehicles, and AI. These successes imply that Chinese scientists and companies have the potential to propose innovative ideas in embodied intelligence and lead the industry's evolution from prototypes to market-ready products.

**Fang:** China possesses a strong foundation for the development of the embodied intelligence industry, including a robust industrial supply chain, abundant data resources, and diverse application scenarios. I am confident that in areas such as intelligent driving, intelligent robots (including industrial robots, rehabilitation robots, and others), as well as multi-agent intelligence, the embodied intelligence industry will experience rapid growth in China.

**Chen:** Amid the wave of embodied intelligence development, we must not only drive technological advancements but also explore innovative application scenarios. We should leverage our imagination and fully utilize the advantages of embodied intelligence in learning, reasoning, and interaction to identify new breakthroughs in industries such as manufacturing, security, services, and disaster response. By doing so, we can realize applications that were previously unimaginable, enabling robots and intelligent systems to better address real-world challenges.

**Ding:** I couldn't agree more. Next step's research of embodied intelligence must be application-oriented. For China to gain international influence in this field, we need to focus on two key areas.

Chinese scientists and companies have the potential to propose innovative ideas in embodied intelligence and lead the industry's evolution from prototypes to market-ready products.

—Yaonan Wang

First, we should try to propose a new scientific framework for intelligent robots. The traditional architecture is no longer suitable, but what will the new framework be like? Will it be end-to-end, or something entirely different? There are significant opportunities for innovation in this direction.

Second, we must strike a balance between cost and performance to achieve breakthroughs in specific applications.

Currently, most notable achievements in intelligent robotics are isolated advancements in specific areas. These breakthroughs have yet to be integrated into a transformative revolution comparable to what we've seen in autonomous driving. The future general intelligent robots that we are pursuing should be able to work flexibly in various industrial and living scenarios, even including unknown and extreme scenarios such as the deep sea and deep space. We still have a long way to go to realize this vision.

I eagerly look forward to seeing more young talents enter this promising and challenging field. They will bring fresh ideas and drive new breakthroughs. I believe the next few years will be a pivotal period for the development of embodied intelligence, with significant breakthroughs in both theoretical and practical application aspects.

## Notes

Weijie Zhao is a news and science editor of NSR based in Beijing. Ye Yuan is a Professor at the School of Mechanical Science and Engineering, Huazhong University of Science and Technology, China.

